# Hints and Suggestions to the Dental Profession

**Published:** 1860-04

**Authors:** T. D. Thompson


### ARTICLE III.

### *Hints and Suggestions to the Dental Profession.* By T. D. Thompson, D. D. S.

It must be a noticeable fact to all properly instructed dentists that a knowledge of physiology, in its most limited sense, is lamentably universal.

Although by far the larger majority of mankind may be in a measure interested in this subject, they nevertheless manifest an indifference to a knowledge of their physical being, which to a reflecting mind, is surprising.

As relates to most of the affairs which affect the welfare of our race we are alive and active.

The love of wealth is so universal to man's nature, that his mind is racked, and his whole being, physical as well as intellectual, taxed to its utmost powers in its pursuit or acquisition.

No ocean is there too broad, no river too long, no mountain so high, as to debar him from this pursuit of *gold.*

Again, of reading, the mind absorbs, maelstrom like, book and pamphlet with an interest to which even the midnight hour is a witness. The matter thus imbibed often is poison to the mental as well as to the physical being.

It surely must be admitted that there is a disregard for "the house I live in," which is hastening its premature ruin and eventual rapid destruction.

The words, "know thyself," are replete with meaning, as relates to man, whether physically or intellectually.

This general deficiency of physical knowledge has often suggested to our mind the *imperative* necessity of every dentist being *well* instructed in the specialties of his profession. Thus instructed, they will be enabled carefully to direct their patients in that department of physiology which is legitimately connected with their professional practice.

Here let me say, that I presume it will be admitted by the majority of those persons who have limited their studies to a dental office, that the incentives have been very feeble to study the various works which treat on anatomy, physiology, and other kindred subjects, necessarily connected with their future professional practice. A want of such knowledge must limit their usefulness in their profession. Skillful manipulations in operative and mechanical dentistry will not always suffice.

I trust the preceding last remarks may meet the notice of some student now pursuing his instructions in a dental office, who has a higher aim than the mere mechanical part of his profession, and who has determined to fit himself fully for his future course in life. Still he may be hesitating as to the necessity, and may question even the value of a course of lectures at a dental college. We would say to such an one, do not hesitate, for we believe that the advantages to be derived from such a course may be of the utmost importance to your future prosperity. Let not the prejudices of others influence your decision. We have never conversed with an individual who had attended a course of lectures at a dental college who did not express himself as being well repaid by the knowledge thus acquired.

Another thought, you will have no after regrets, but will feel that you have by thus doing aided in the advancement and elevation of the profession of your choice.

